# Genetic deletion of Polo-like kinase 2 reduces alpha-synuclein serine-129 phosphorylation in presynaptic terminals but not Lewy bodies

**DOI:** 10.1016/j.jbc.2021.100273

**Published:** 2021-01-09

**Authors:** Leah J. Weston, Teresa L. Stackhouse, Kateri J. Spinelli, Sydney W. Boutros, Elizabeth P. Rose, Valerie R. Osterberg, Kelvin C. Luk, Jacob Raber, Tamily A. Weissman, Vivek K. Unni

**Affiliations:** 1 Department of Neurology & Jungers Center for Neurosciences Research, Oregon Health & Science University, Portland, Oregon, USA; 2 Department of Behavioral Neuroscience, Oregon Health & Science University, Portland, Oregon, USA; 3 Neuroscience Graduate Program, Vollum Institute, Oregon Health & Science University, Portland, Oregon, USA; 4 Department of Pathology and Laboratory Medicine and Center for Neurodegenerative Disease Research, University of Pennsylvania Perelman School of Medicine, Philadelphia, Pennsylvania, USA; 5 Departments of Behavioral Neuroscience, Neurology, and Radiation Medicine and Division of Neuroscience, ONPRC, Oregon Health & Science University, Portland, Oregon, USA; 6 Department of Biology, Lewis & Clark College, Portland, Oregon, USA; 7 OHSU Parkinson Center, Oregon Health & Science University, Portland, Oregon, USA

**Keywords:** Polo-like kinase 2, alpha-synuclein, Lewy body, presynaptic terminal, CK, casein kinase, DLB, Dementia with Lewy bodies, DSB, double-strand break, FRAP, fluorescence recovery after photobleaching, GFP, green fluorescent protein, IHC, immunohistochemistry, PD, Parkinson’s disease, PFF, preformed fibril, PLK, Polo-like kinase, WT, wild-type

## Abstract

Phosphorylation of alpha-synuclein at serine-129 is an important marker of pathologically relevant, aggregated forms of the protein in several important human diseases, including Parkinson’s disease, Dementia with Lewy bodies, and Multiple system atrophy. Although several kinases have been shown to be capable of phosphorylating alpha-synuclein in various model systems, the identity of the kinase that phosphorylates alpha-synuclein in the Lewy body remains unknown. One member of the Polo-like kinase family, PLK2, is a strong candidate for being the Lewy body kinase. To examine this possibility, we have used a combination of approaches, including biochemical, immunohistochemical, and *in vivo* multiphoton imaging techniques to study the consequences of PLK2 genetic deletion on alpha-synuclein phosphorylation in both the presynaptic terminal and preformed fibril-induced Lewy body pathology in mouse cortex. We find that PLK2 deletion reduces presynaptic terminal alpha-synuclein serine-129 phosphorylation, but has no effect on Lewy body phosphorylation levels. Serine-129 mutation to the phosphomimetic alanine or the unphosphorylatable analog aspartate does not change the rate of cell death of Lewy inclusion-bearing neurons in our *in vivo* multiphoton imaging paradigm, but PLK2 deletion does slow the rate of neuronal death. Our data indicate that inhibition of PLK2 represents a promising avenue for developing new therapeutics, but that the mechanism of neuroprotection by PLK2 inhibition is not likely due to reducing alpha-synuclein serine-129 phosphorylation and that the true Lewy body kinase still awaits discovery.

Parkinson’s disease (PD) is a progressive neurological disorder with a variety of motor and nonmotor symptoms affecting approximately 10 million people worldwide (1). The motor symptoms are due to dopamine neuron loss in the substantia nigra of the midbrain (2). Cytoplasmic inclusions called Lewy bodies are observed in surviving dopamine neurons, and Lewy bodies in other peripheral and central nervous system regions correlate with many of the nonmotor symptoms of PD (3). In addition, a group of related disorders termed “synucleinopathies” are characterized by Lewy body presence in a variety of nondopaminergic neuron types that is also associated with neurological dysfunction, such as cortical Lewy bodies and cognitive dysfunction in Dementia with Lewy bodies (DLB), or autonomic system Lewy bodies and orthostatic hypotension in pure autonomic failure. Lewy bodies are somatic structures comprised of misfolded and aggregated alpha-synuclein, which is normally localized primarily to the presynaptic terminal and nucleus (4, 5).

One of the characteristic hallmarks of aggregated alpha-synuclein within Lewy bodies is a high level of specific posttranslational modifications of the protein, including phosphorylation, ubiquitination, nitrosylation, and C-terminal truncation (6). Among these possible posttranslational modifications of alpha-synuclein, understanding the role of phosphorylation at serine-129 has generated significant interest in the field. This is in part because on a molar basis serine-129 phosphorylation is the most abundant posttranslational modification found in Lewy pathology alpha-synuclein: >90% of Lewy inclusion alpha-synuclein is phosphorylated at this site, while <4% of normal brain alpha-synuclein contains this modification (6). In addition, serine-129 phosphorylation appears to be one of the most sensitive immunohistochemical markers developed for detecting disease-associated pathological alpha-synuclein in human autopsy tissue (7). Phosphorylation also provides an attractive therapeutic drug target for potentially modulating PD progression (8), since kinase inhibition has proven to be an effective strategy for developing therapeutics in many diseases. It is still unclear, however, whether serine-129 phosphorylation directly mediates neurotoxicity or not. Several groups have shown little to no role, or even beneficial effects, due to this phosphorylation (9, 10, 11, 12, 13). In contrast, others have argued that decreasing alpha-synuclein serine-129 phosphorylation ameliorates neurodegeneration (14, 15, 16, 17, 18, 19, 20).

Several different serine/threonine kinases have been shown to be capable of phosphorylating synuclein family members. For instance, calcium/calmodulin-dependent protein kinase II can phosphorylate beta- and gamma-synuclein, but not alpha-synuclein, in *in vitro* assays (21, 22). In contrast, several different kinase families, including G-protein-coupled receptor kinase (GRK, (22)), casein kinase (CK, (22)), and Polo-like kinase (PLK, (23)), can catalyze alpha-synuclein serine-129 phosphorylation *in vitro*. Specifically, GRK family members GRK2 and GRK5 can phosphorylate purified, soluble alpha-synuclein at this site (22), as can CK1 and CK2 (22, 24, 25) and PLK1, PLK2, and PLK3 (23, 26). In cell culture, all three kinase families GRK (22, 27), CK (24, 25) and PLK (23, 26, 28, 29) can phosphorylate soluble alpha-synuclein at serine-129, but only PLK phosphorylates aggregated alpha-synuclein forms (26, 28). Furthermore, in mouse and rat brain PLK family members, including PLK2, play a major role in serine-129 phosphorylation (23, 26, 28, 30, 31, 32), suggesting that PLK2 may be important for Lewy body phosphorylation and/or formation. To the best of our knowledge, however, it has not yet been tested whether PLK2 phosphorylates alpha-synuclein specifically in Lewy bodies or how PLK2 activity might regulate Lewy body formation.

In order to test the role of PLK2 as a Lewy body alpha-synuclein kinase, we used a combination of approaches, including genetic deletion of PLK2 and transgenic mouse models amenable to *in vivo* multiphoton imaging. Specifically, we used transgenic mice that express wild-type human sequence alpha-synuclein tagged on its C-terminus with enhanced green fluorescent protein (Syn-GFP) (33) and intracortical injection of alpha-synuclein preformed fibrils (PFFs) to induce Lewy pathology in Syn-GFP animals as we previously reported (34). This approach has several advantages, including the ability to study phosphorylation of human sequence protein, the ability to measure alpha-synuclein aggregation in distinct subcellular compartments, and the ability to study the consequences of this aggregation in identified neurons *in vivo* over a period of months using our established multiphoton imaging paradigm in cortex (34, 35, 36, 37). Using these approaches, we show that PLK2 contributes to serine-129 phosphorylation of alpha-synuclein at presynaptic terminals, but that its genetic deletion does not change levels of somatic Lewy pathology phosphorylation. In contrast, PLK2 deletion does increase survival of individual neurons bearing Lewy inclusions. By using point mutations to study the effect of serine-129 phosphorylation, we show that this protective effect of PLK2 deletion is likely not due to specific effects on this phosphorylation, but rather to other consequences of PLK2 deletion.

## Results

**Figure 1:**
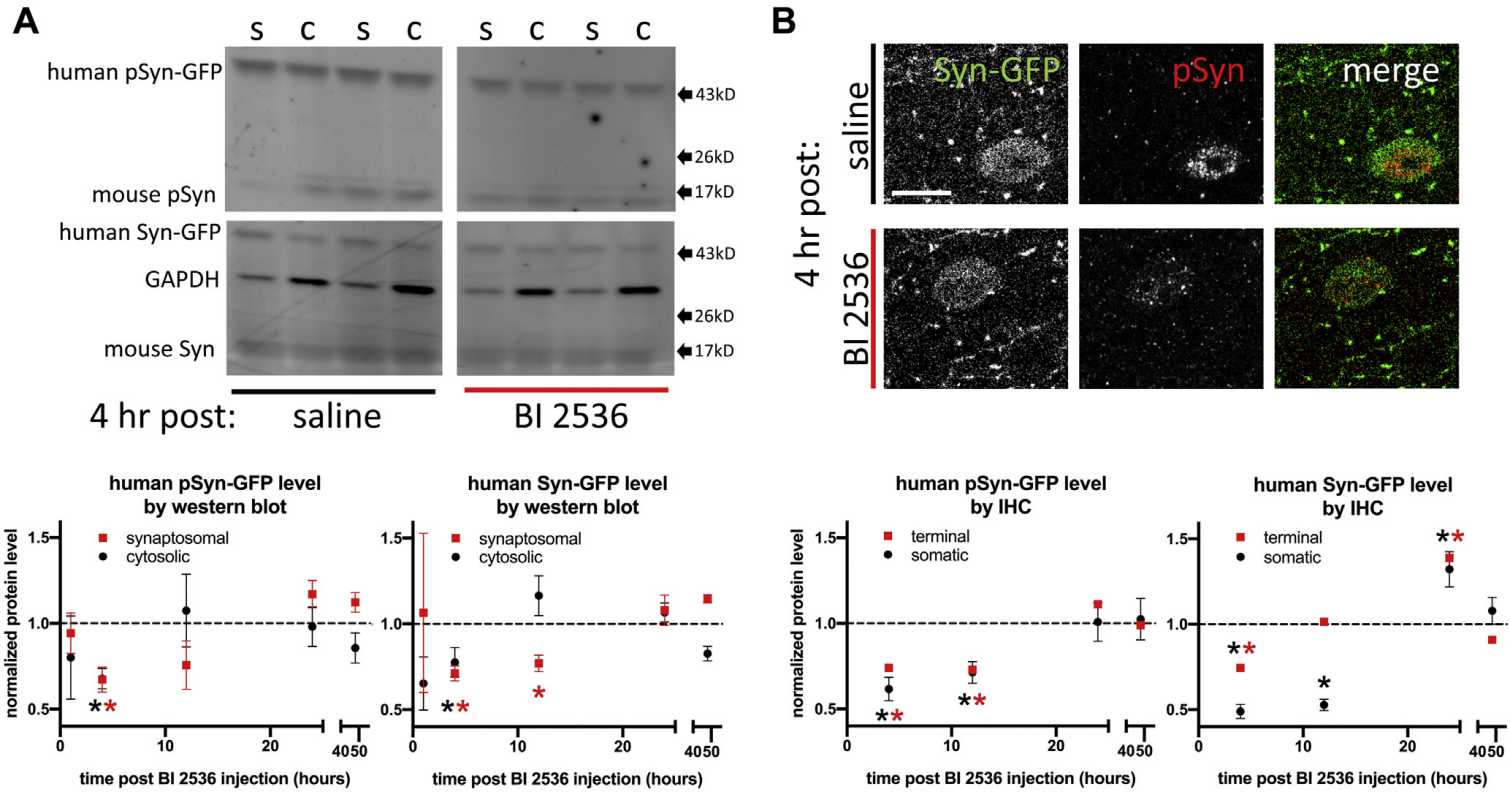
**Polo-like kinase inhibition with BI 2536 acutely decreases presynaptic alpha-synuclein serine-129 phosphorylation and total protein levels.***A*, Top: Western blot of Syn-GFP mouse brain separated into synaptosomal (s) and cystosolic (c) fractions and stained for human serine-129 phospho-Syn-GFP (pSyn-GFP) and total Syn-GFP 4 h after either saline or BI 2536 IP injection. GAPDH is used as a loading control and endogenous mouse pSyn and total Syn bands are also shown, although the endogenous mouse proteins were not quantified because of their relatively low signal. Bottom: Synaptosomal and cystosolic pSyn-GFP and Syn-GFP levels quantified at times from 1 to 48 h after saline and BI 2536 injection. Each time point represents the value of BI 2536 samples normalized to matched saline controls for that time point. A significant decrease in pSyn-GFP was only seen at 4 h postinjection (synaptosomal: mean = 0.672 ± 0.074, 95% CI = 0.483–0.861; cystosolic: mean = 0.678 ± 0.059, 95% CI = 0.526–0.829) and for Syn-GFP at 4 h for both synaptosomal and cytosolic fractions and 12 h for only the synaptosomal fraction (4 h synaptosomal: mean = 0.712 ± 0.044, 95% CI = 0.599–0.825; 12 h synaptosomal: mean = 0.770 ± 0.048, 95% CI = 0.616–0.923; 4 h cytosolic: mean = 0.776 ± 0.086, 95% CI = 0.554–0.998). N=3–6 animals per time point per treatment condition. *B*, Top: Fixed tissue histological staining from Syn-GFP mouse cortex shows one Syn-GFP-positive neuron and many smaller Syn-GFP-positive presynaptic terminal puncta by endogenous GFP fluorescence and pSyn immunostaining 4 h after either saline or BI 2536 IP injection. Scale bar 10 μm. Bottom: Presynaptic terminal and somatic pSyn-GFP and Syn-GFP fluorescence level quantified at times from 1 to 48 h after saline and BI 2536 injection. Each time point represents the value of BI 2536 levels normalized to matched saline controls for that time point. A significant decrease in pSyn-GFP was seen at 4 h (terminal: mean = 0.740 ± 0.022, 95% CI = 0.691–0.788; somatic: mean = 0.617 ± 0.068, 95% CI = 0.479–0.756) and 12 h (terminal: mean = 0.729 ± 0.032, 95% CI = 0.658–0.799; somatic: mean = 0.714 ± 0.063, 95% CI = 0.586–0.841) postinjection and for Syn-GFP a significant decrease was detectd at 4 h (terminal: mean = 0.743 ± 0.052, 95% CI = 0.629–0.857; somatic: mean = 0.489 ± 0.037, 95% CI = 0.405–0.574) and 12 h (somatic: mean = 0.527 ± 0.087, 95% CI = 0.461–0.594) and an increase at 24 h (terminal: mean = 1.388 ± 0.042, 95% CI = 1.295–1.481; somatic: mean = 1.322 ± 0.104, 95% CI = 1.111–1.533) postinjection. For terminals N = 12 regions of interest/3–4 animals per time point per treatment condition; for somata N = 24–45 cells/3–4 animals per time point per treatment condition.

To test the role of PLK family members in the phosphorylation of alpha-synuclein at serine-129 in both presynaptic terminal and other cytosolic subcellular compartments within cortical neurons, we treated heterozygous Syn-GFP transgenic animals with a single dose of the selective small-molecule PLK inhibitor BI 2536 (50 mg/kg) and measured phosphorylated and total Syn-GFP levels in cortical tissue using western blot analysis after biochemical fractionation. We determined the time course of the resulting phosphorylation changes by sacrificing animals at various times following BI 2536 injection. Both synaptosomal and cytosolic pools of Syn-GFP showed significant decreases in phosphorylated and total levels starting at 4 h after BI 2536 administration, which then returned to baseline levels by 12 h and remained there at 24 and 48 h (Fig. 1*A*). An analysis of Syn-GFP animals similarly treated with BI 2536 using immunohistochemistry (IHC) for phosphorylated Syn-GFP and GFP fluorescence signal for measuring total levels from cortical sections revealed similar results. Significant decreases in presynaptic and somatic levels were again observed 4 h after BI 2536 administration (Fig. 1*B*). Phosphorylated Syn-GFP levels remained reduced at 12 h but returned to baseline by 24 h, while total levels recovered to baseline in terminals and remained reduced in the cell body at 12 h (Fig. 1*B*). At 24 h post BI 2536 administration, both terminal and somatic total Syn-GFP levels were transiently increased before returning to baseline at 48 h (Fig. 1*B*). Since BI2536 is known to inhibit PLK1, PLK2, and PLK3 (38, 39, 40), these experiments suggest that endogenous mouse PLK constitutively phosphorylates cortical Syn-GFP in this transgenic line.

**Figure 2:**
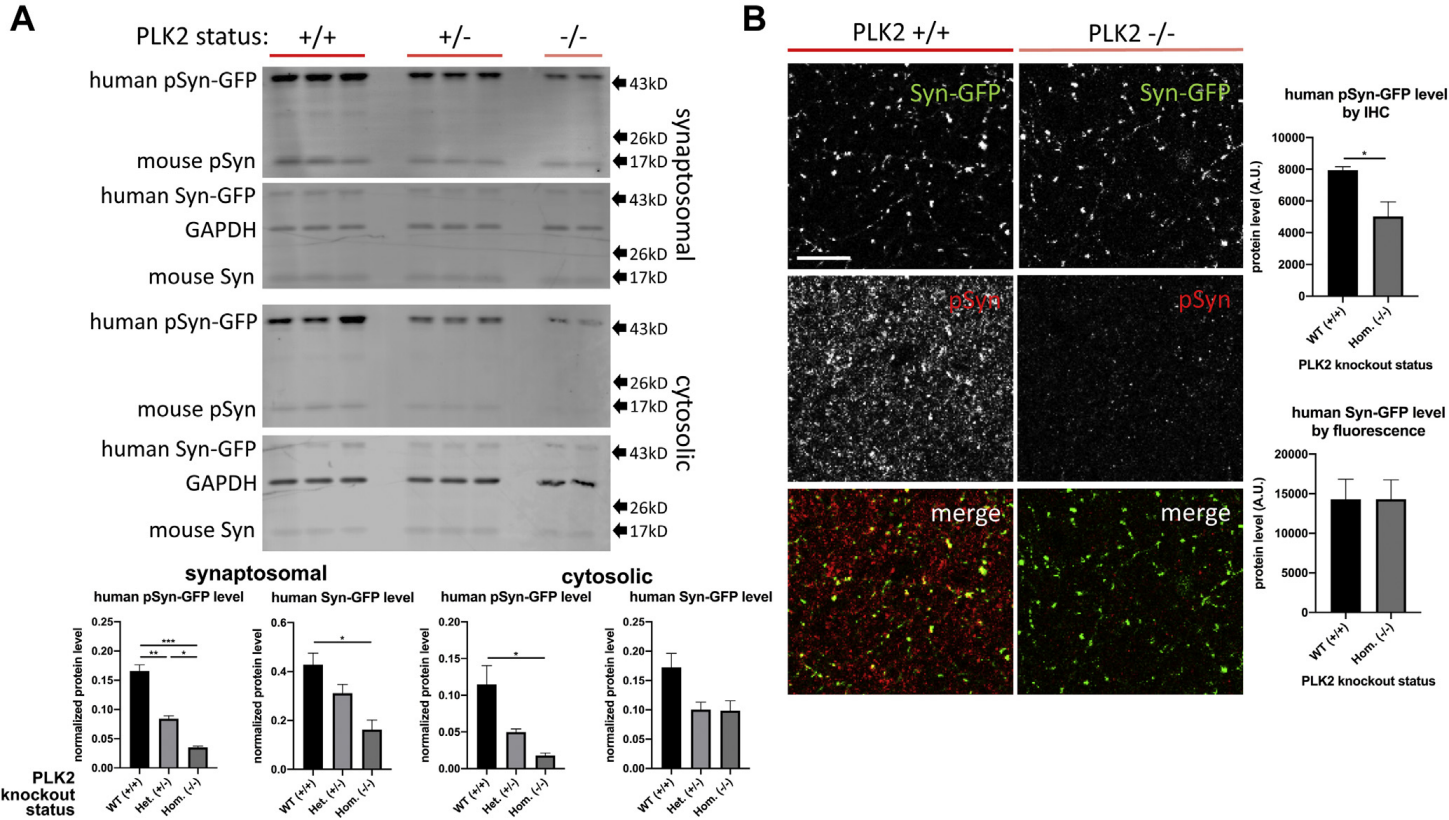
**Polo-like kinase 2 genetic deletion decreases presynaptic alpha-synuclein serine-129 phosphorylation.***A*, Top: Western blot of Syn-GFP x PLK2 knockout mouse brain synaptosomal (above) and cytosolic (below) fractions stained for serine-129 phospho-Syn-GFP (pSyn-GFP), total Syn-GFP (Syn-GFP), and a GAPDH loading control (used for normalization) in PLK2 wild-type (WT, +/+), heterozygous knockout (Het. +/-), and homozygous knockout (Hom. −/−) animals. Endogenous mouse pSyn and total Syn bands are also shown, although the endogenous mouse proteins were not quantified because of their relatively low signal. For synaptosomal fractions, significant stepwise decreases in mean pSyn-GFP levels were seen between WT, Het., and Hom. animals (WT = 0.166 ± 0.011, Het.=0.084 ± 0.004, Hom.=0.033 ± 0.002); one-way ANOVA (F(2, 5) = 67.71, *p* = 0.0002); post-hoc Tukey tests: WT vs. Het. *p* = 0.0013, WT vs. Hom. *p* = 0.0002, Het. vs. Hom. *p* = 0.0189. A significant decrease in mean Syn-GFP level was seen between WT and Hom. animals (WT = 0.428 ± 0.047, Het.=0.311 ± 0.036, Hom.=0.163 ± 0.039); one-way ANOVA (F(2, 5) = 8.809, *p* = 0.0230); post-hoc Tukey tests: WT vs. Het. *p* = 0.1910, WT vs. Hom. *p* = 0.0194, Het. vs. Hom. *p* = 0.1394. For cytosolic fractions, a significant decrease in mean pSyn-GFP levels was seen between WT and Hom. animals (WT = 0.115 ± 0.023, Het.=0.050 ± 0.004, Hom.=0.018 ± 0.003); one-way ANOVA (F(2, 5) = 7.626, *p* = 0.0303); post-hoc Tukey tests: WT vs. Het. *p* = 0.0850, WT vs. Hom. *p* = 0.0314, Het. vs. Hom. *p* = 0.4921. No significant differences were seen in Syn-GFP levels between groups (WT = 0.172 ± 0.024, Het.=0.100 ± 0.013, Hom.=0.099 ± 0.017); one-way ANOVA (F(2, 5) = 4.958, *p* = 0.0651); post-hoc Tukey tests: WT vs. Het. *p* = 0.0829, WT vs. Hom. *p* = 0.1095, Het. vs. Hom. *p* = 0.9986. N = 2–3 animals per group. *B*, Left: Fixed tissue histological staining from Syn-GFP mouse cortex shows numerous Syn-GFP-positive presynaptic terminal puncta by endogenous GFP fluorescence and pSyn immunostaining in PLKW2 WT and Hom. animals. Scale bar 10 μm. Rigth: A significant decrease in mean presynaptic terminal pSyn-GFP immunofluorescence was seen between WT and Hom. animals (WT = 7936 ± 229, Hom.=5020 ± 926); unpaired *t*-test, two-tailed *p* = 0.0302). No significant difference was seen in mean presynaptic terminal Syn-GFP fluorescence (WT = 14,283 ± 2547, Hom.= 14,297 ± 2462); unpaired *t*-test, two-tailed *p* = 0.9973). N = 2–3 animals per group.

To be able to more directly test the role of PLK in the phosphorylation of alpha-synuclein in mouse brain, we bred our Syn-GFP line to PLK2 knockout (PLK2 KO) animals to generate heterozygous Syn-GFP mice that were on a PLK2 wild-type (WT, +/+), heterozygous KO (+/-), or homozygous KO (−/−) background. We chose the PLK2 family member because several independent reports suggest that PLK2 is a major synuclein kinase *in vitro* and in mouse, rat, and human Lewy body disease brain (23, 26, 28, 30, 31, 32). Consistent with our pharmacological inhibition of PLK, genetic deletion of PLK2 caused a decrease in phosphorylated Syn-GFP in both the synaptosomal and cytosolic fractions generated from cortical tissue, as determined by western blot analysis (Fig. 2*A*). Total Syn-GFP levels were also decreased in PLK2 KO synaptosomes, but not in the cytosolic fraction (Fig. 2*A*). A parallel analysis of cortical tissue by IHC demonstrated a significant decrease in phosphorylated, but not total, Syn-GFP in the cortical presynaptic terminals of PLK2 KO mice (Fig. 2*B*). These data suggest that PLK2 is important for phosphorylation of alpha-synuclein at terminals and in the cytosol and that this may regulate total alpha-synuclein levels, as has been suggested previously (41, 42). The exact mechanism by which this regulation of levels occurs, however, is subject to some controversy. It has been reported that PLK2-dependent alpha-synuclein phosphorylation can promote a PLK2–synuclein complex to form that targets synuclein for clearance by the autophagy-lysosomal pathway (41). Alternatively, others have suggested that PLK2 regulates expression *via* a mechanism that is independent of PLK2-mediated alpha-synuclein phosphorylation and which decreases transcription from the alpha-synuclein (SNCA) gene (42).

**Figure 3:**
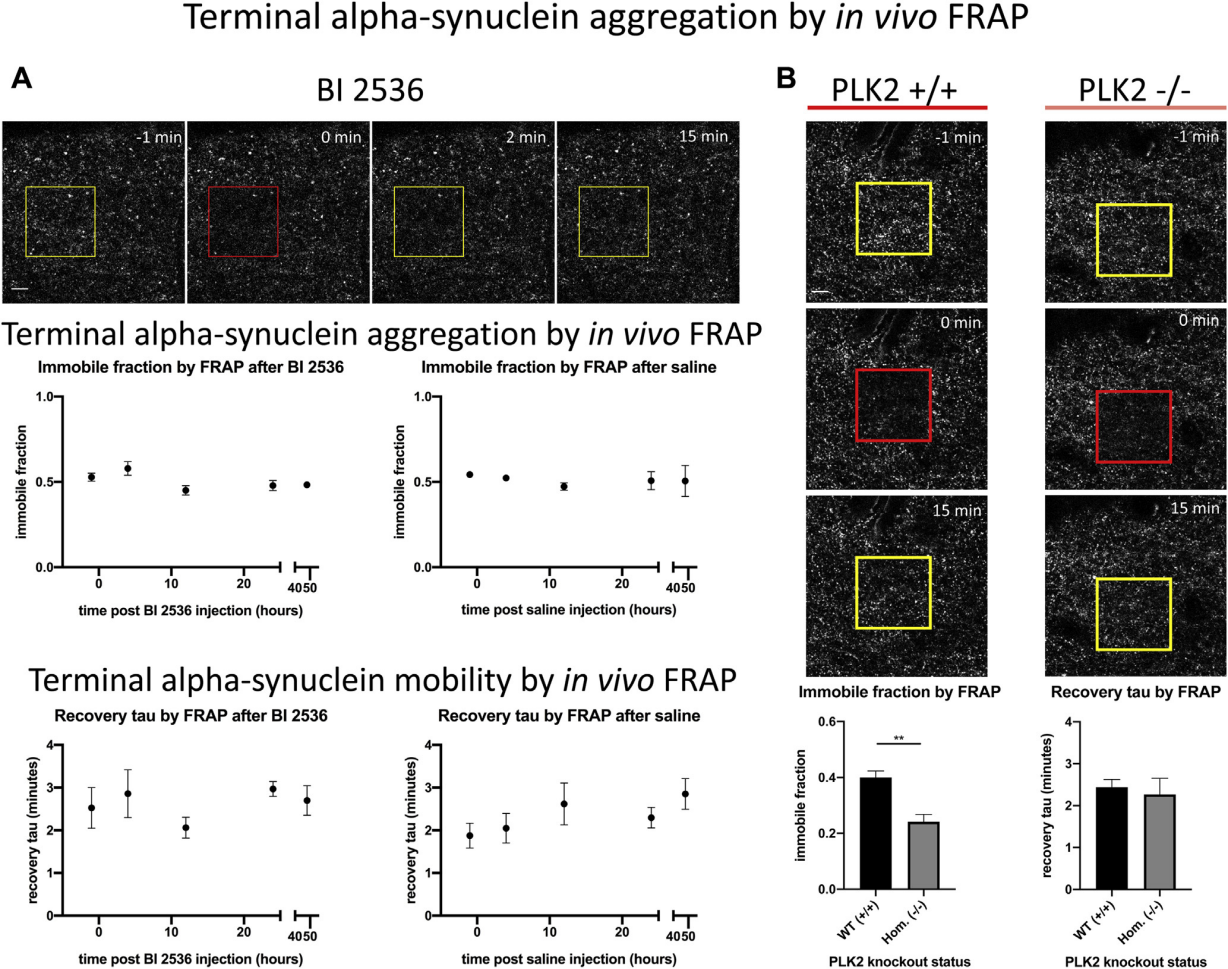
**Polo-like kinase inhibition with BI 2536 does not alter presynaptic aggregation while Polo-like kinase 2 genetic deletion does reduce *in vivo* measures of aggregation.***A*, Top: *In vivo* multiphoton imaging from cortex in a Syn-GFP mouse shows region of interest labeled with numerous Syn-GFP-positive presynaptic terminal puncta. Region of bleach for FRAP experiments shown within yellow square before, red square immediately after, and yellow square at various times after the bleach pulse. Scale bar 10um. Middle: The calculated immobile fraction of presynaptic terminal Syn-GFP at 15 min postbleach before and after BI 2536 or saline injection (given at time point 0) shows no significant differences at any time point. Bottom: The calculated recovery tau similarly shows no significant differences at any time point. N = 4–10 regions of interest/4–6 animals per time point. *B*, Top: *In vivo* multiphoton imaging from cortex in a Syn-GFP mouse shows region of interest labeled with numerous Syn-GFP-positive presynaptic terminal puncta. Region of bleach for FRAP experiments shown within yellow square before, red square immediately after, and yellow square at a time point after the bleach pulse. Scale bar 10 μm. Bottom: The calculated immobile fraction of presynaptic terminal Syn-GFP at 15 min postbleach showed a significant decrease between WT and Hom. animals (WT = 0.400 ± 0.023, Hom.=0.242 ± 0.026; unpaired *t*-test, two-tailed *p* = 0.0015). The calculated recovery tau shows no significant differences between WT and Hom. animals (WT = 2.441 ± 0.186 min, Hom.=2.269 ± 0.382 min); unpaired *t*-test, two-tailed *p* = 0.6575).

In order to test whether changes in presynaptic terminal alpha-synuclein phosphorylation had effects on its aggregation in this cellular compartment, we used fluorescence recovery after photobleaching (FRAP) approaches that we have previously developed to study presynaptic Syn-GFP aggregation in mouse cortex using *in vivo* multiphoton imaging (Unni *et al*., 2010; Spinelli *et al*., 2014, 2015). We first measured the potential effect of PLK inhibition and reduced Syn-GFP serine-129 phosphorylation on this protein’s terminal aggregation using the BI 2536 inhibitor. We did not detect any differences in either the fraction of terminal Syn-GFP that was immobile (immobile fraction, IF) or the FRAP recovery time constant (tau) 1–48 h after BI 2536 administration (Fig. 3*A*), suggesting that there was no change in terminal Syn-GFP aggregation or membrane binding caused by this level of phosphorylation reduction. We next used similar *in vivo* multiphoton FRAP approaches to test whether genetic deletion of PLK2 altered terminal alpha-synuclein aggregation. These studies revealed a decrease in the IF in PLK2 KO mice, but no change in the recovery tau (Fig. 3*B*). Based on our previous work (Spinelli *et al*., 2014), this indicates that PLK2 deletion does not alter Syn-GFP binding to synaptic vesicle membranes *in vivo*, but does reduce the amount of aggregated Syn-GFP protein in cortical terminals. We next performed an analysis of several measures of cognitive and motor performance in Syn-GFP/PLK2 WT and KO mice to determine if the decreased level of terminal alpha-synuclein aggregation in PLK2 KO mice we measured with our *in vivo* imaging paradigm was associated with any changes in behavioral performance. We detected very few significant differences in open field activity, novel object recognition, rotorod, Morris water maze, contextual or cued fear conditioning performance between the two groups (Fig. S1).

**Figure 4:**
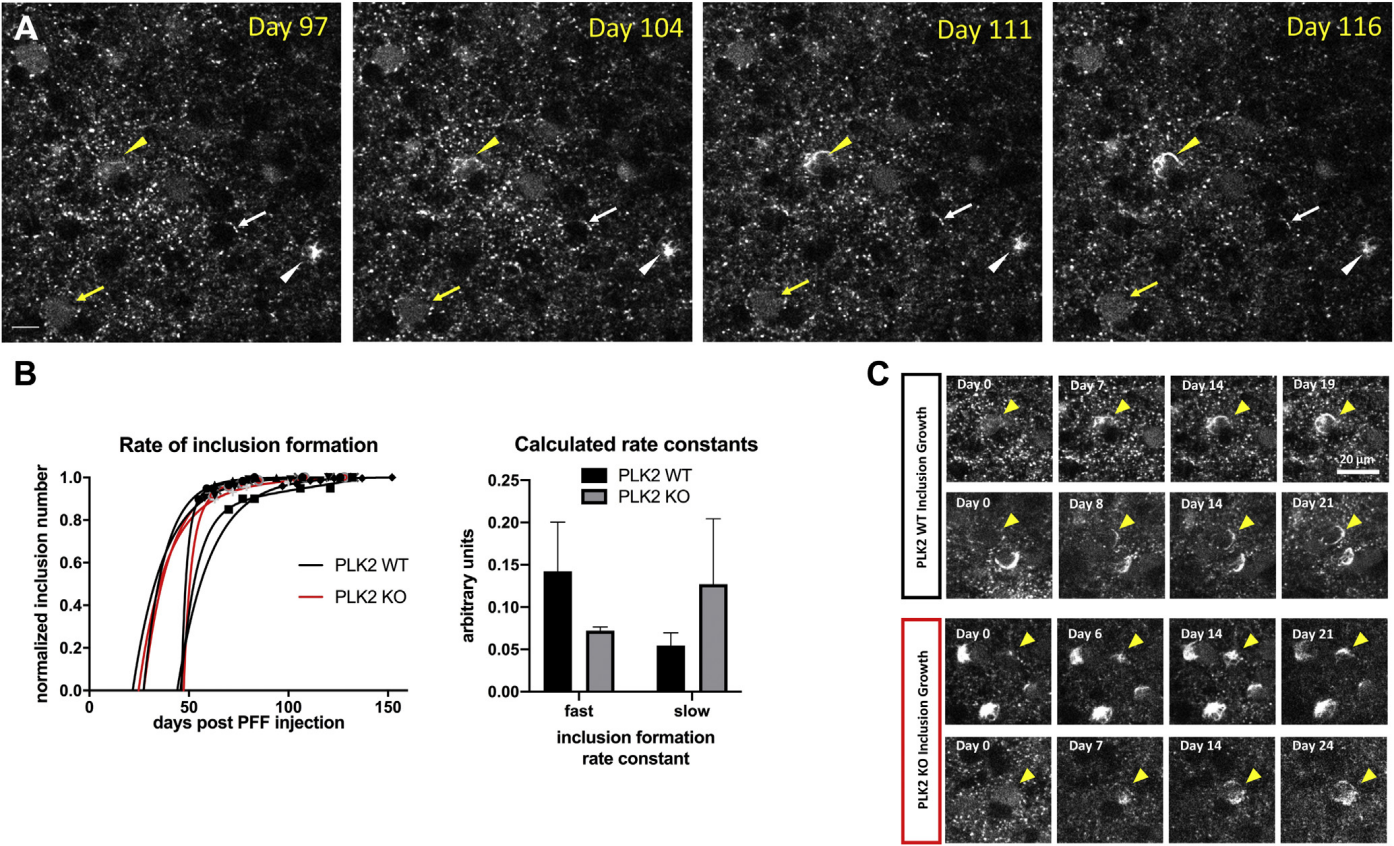
**Polo-like kinase 2 genetic deletion does not alter rate of PFF-induced Lewy inclusion formation.***A*, *In vivo* multiphoton imaging from cortex in a Syn-GFP mouse at the days indicated after PFF injection showed formation of a new Lewy inclusion (*yellow arrowhead*) over the course of 19 days. A previously formed inclusion (*white arrowhead*), and examples of neurons expressing unaggregated Syn-GFP (*yellow arrow*) or no Syn-GFP (*white arrow*) are shown. Scale bar 10 μm. *B*, Left: The number of inclusions present in specific regions of interest over time, starting approximately 50 days after PFF injection, showed new formation essentially ending by approximately 100 days post PFF injection. Right: Calculated fast and slow rate constants for inclusion formation using a double exponential model showed no significant difference between PLK2 WT and KO animals (fast rate constant: WT = 0.142 ± 0.058, KO = 0.072 ± 0.004, unpaired *t*-test, two-tailed *p* = 0.3990; slow rate constant: WT = 0.055 ± 0.015, KO = 0.127 ± 0.077, unpaired *t*-test, two-tailed *p* = 0.2707; N = 52–664 inclusions per animal/3–5 animals per group). *C*, The growth of individual inclusions took approximately 2–3 weeks in both PLK2 WT and KO animals. Scale bar 20 μm.

In previous work, we have shown that intracortical injection of recombinantly generated and *in vitro* aggregated alpha-synuclein PFFs into Syn-GFP mice produced robust somatic Lewy pathology composed of the GFP-tagged protein (34). In order to directly test the role of PLK2 genetic deletion on the process of Lewy pathology formation, we next used our established PFF injection paradigm in Syn-GFP mice on a PLK WT and KO background. Imaging using *in vivo* multiphoton microscopy showed the clear formation of somatic Syn-GFP Lewy pathology in cortical neurons (Fig. 4*A*, Fig. S2*A*), similar to our previous results (34). The rate of formation of new inclusions starting ∼50–60 days after PFF injection (the first time point that we were able to do *in vivo* multiphoton imaging) was well fit with a double exponential model and showed no significant differences in the formation rate between PLK2 WT and KO animals of either the fast or slow rate constants (Fig. 4*B*). We were able to occasionally follow the formation of Lewy pathology within individual cortical neurons *in vivo* in PLK2 WT and KO mice, although the number of such neurons was limited since the probability of our imaging any one particular cell during the formation event is low. In the observed cases, the time to form a complete Lewy inclusion within a neuron was ∼2–3 weeks and was similar in both PLK2 WT and KO animals (Fig. 4*C*). Next, we measured the aggregation level of individual Syn-GFP Lewy inclusions in PLK2 WT and KO animals using *in vivo* multiphoton FRAP, as we have previously done (34). These data show that almost all Syn-GFP protein in somatic Lewy inclusion-containing neurons is aggregated, with no significant differences between inclusions studied in PLK2 WT and KO animals (Fig. S2*B*). These results suggest that PLK2 activity is not critical for Lewy body formation or level of aggregation.

**Figure 5:**
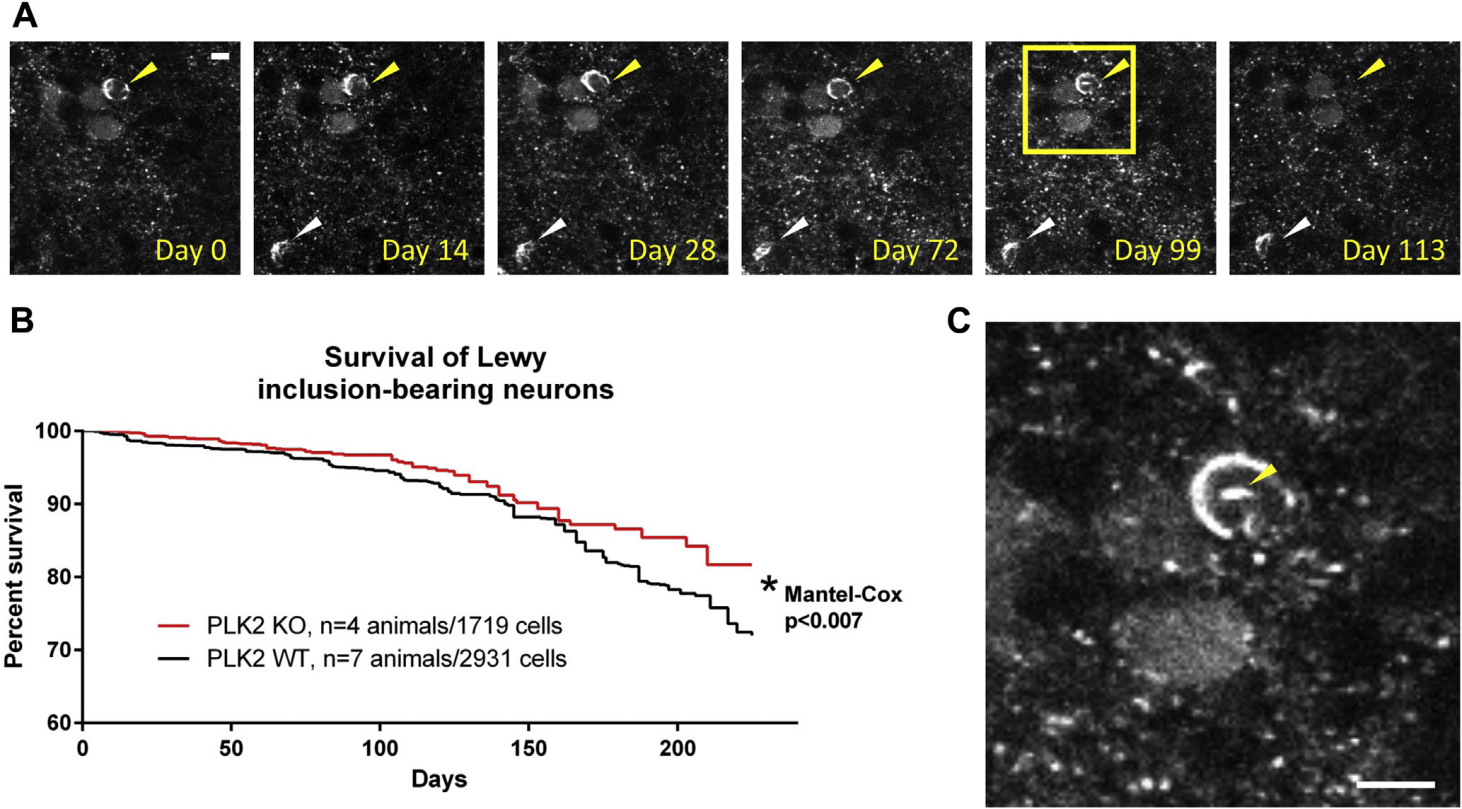
**Polo-like kinase 2 genetic deletion protects neurons from degeneration.***A*, Examples of Syn-GFP Lewy inclusion-bearing neurons (*arrowheads*) followed *in vivo* in mouse cortex over 113 days, one of which (*yellow arrowhead*) degenerates between 99 and 113 days. Scale bar 5 μm. *B*, Group data shows significant protection against cell death of Syn-GFP inclusion-bearing neurons on the PLK2 KO background. *C*, Larger image from Day 99 in A (*yellow square*) showing the development of a potentially intranuclear satellite inclusion (*yellow arrowhead*) preceding cell death. Scale bar 8 μm.

We next wanted to test whether PLK2 activity influences the cell death of Lewy inclusion-bearing neurons over time. We therefore followed individual cortical neurons after they had formed somatic Lewy inclusions in Syn-GFP/PLK2 WT and KO animals using *in vivo* multiphoton imaging for ∼230 days. Previously, we demonstrated that only cells containing Syn-GFP Lewy inclusions are destined to die in this time frame and that they do so with a half-time of ∼180 days after formation of the inclusion (34). Here we found a small increase in long-term survival of cortical neurons bearing Syn-GFP Lewy inclusions on the PLK2 KO background as compared with PLK2 WT animals, suggesting that deletion of PLK2 conferred some relative protection from neuronal death (Fig. 5). Our imaging data also demonstrated that at times we could detect a subtle change in the Lewy inclusion morphology that occurred a few days to weeks before its ultimate demise. This change was the development of a new, smaller “satellite” Syn-GFP inclusion within the cell that did not appear to be connected to the preexisting, larger somatic inclusion (Fig. 5*C*). Often, this satellite inclusion appeared to be located within the spherical nucleus of the inclusion-bearing neuron. The number of these events was relatively small, so it was not possible to compare potential differences in these satellite inclusions between PLK2 WT and KO animals. In order to test that the cell death that we observed using our *in vivo* multiphoton imaging techniques was not artifactually caused or accelerated by cumulative phototoxicity due to the imaging process itself, we performed a control imaging experiment in Syn-GFP/PLK2 WT animals. We imaged one cohort with our usual frequency of every 3–14 days (total of 17 imaging sessions) and in the other cohort we only performed three imaging sessions. Both cohorts were imaged over a total ∼160-day period after formation of Lewy inclusions. An analysis of Lewy inclusion-bearing cortical neurons with these two imaging frequencies showed no significant difference in the measured rates of cell death after ∼160 days (Fig. S3), indicating that the cell death we measure with our paradigm is unlikely to be artifactually caused by phototoxicity from multiphoton illumination.

**Figure 6:**
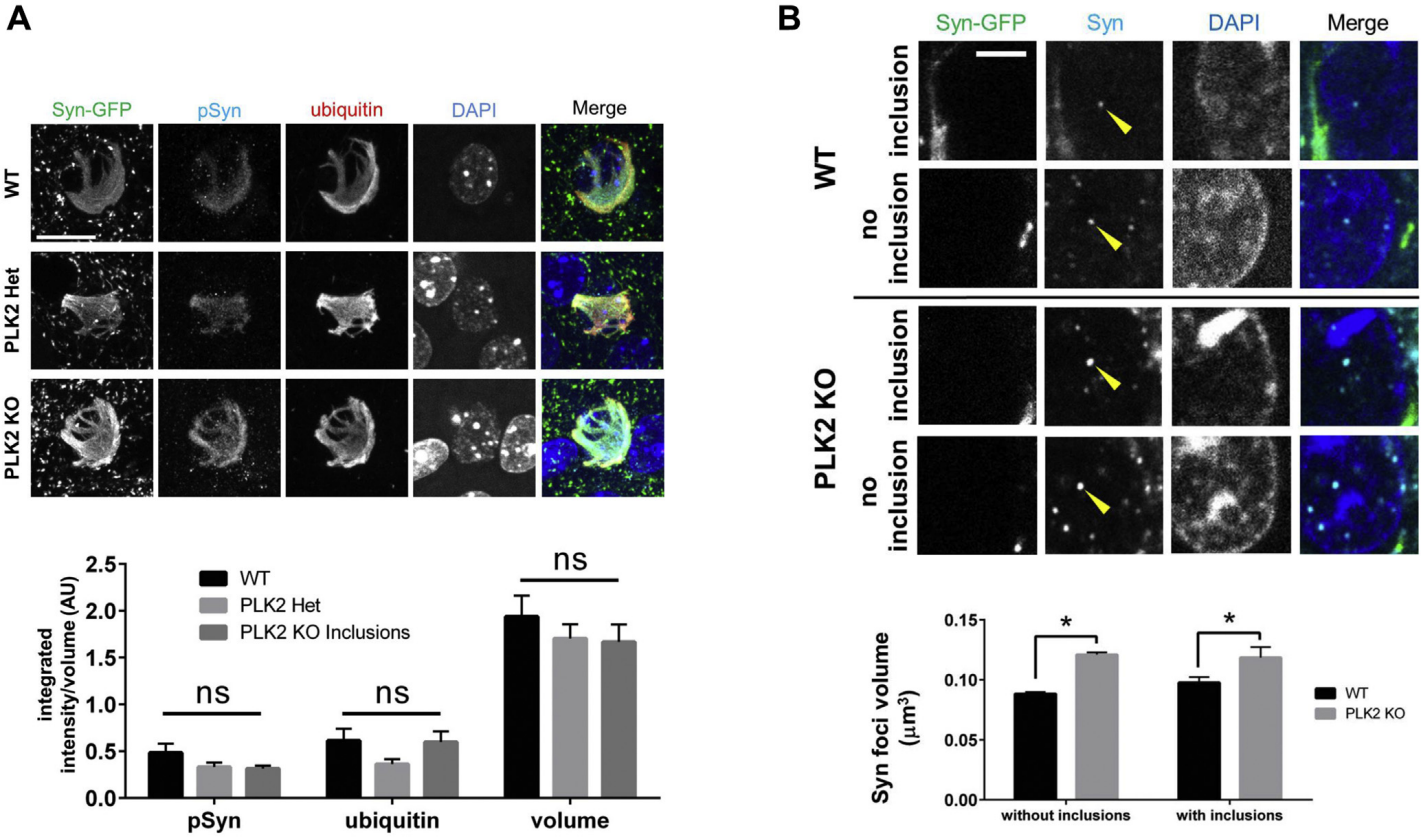
**Polo-like kinase 2 deletion does not reduce Lewy pathology phosphorylation, ubiquitination, or volume, but does increase nuclear alpha-synuclein foci volume.***A*, Staining for cytoplasmic Syn-GFP Lewy inclusion serine-129 phosphorylated alpha-synuclein (pSyn), ubiquitin staining, and measurements of inclusion volume show no significant difference between WT, PLK2 heterozygous deletion, or PLK2 KO (pSyn: WT 0.485 ± 0.095, N = 17 inclusions/4 animals, PLK2 Het. 0.332 ± 0.047, N = 14 inclusions/4 animals, PLK2 KO 0.315 ± 0.033, N = 39 inclusions/4 animals, one-way ANOVA *p* > 0.0769; ubiquitin: WT 0.613 ± 0.128, N = 17 inclusions/4 animals, PLK2 Het. 0.363 ± 0.053, N = 14 inclusions/4 animals, PLK2 KO 0.599 ± 0.114 N = 39 inclusions/4 animals, one-way ANOVA *p* > 0.40; volume: WT 1.94 ± 0.22 x10^2^ μm^3^, N = 30 inclusions/4 animals, PLK2 Het. 1.71 ± 0.15 x10^2^ μm^3^, N = 21 inclusions/4 animals, PLK2 KO 1.67 ± 0.19 x10^2^ μm^3^, N = 30 inclusions/4 animals, one-way ANOVA *p* > 0.55). Scale bar 5 μm. *B*, Measurements of nuclear alpha-synclein foci volume in PLK2 KO animals are significantly larger than WT in neurons with and without Lewy inclusions (without inclusions: WT Syn foci volume 0.0883 ± 0.0016 μm^3^ N = 5199 nuclei/4 animals, PLK2 KO Syn foci volume 0.1209 ± 0.0020 μm^3^ N = 5419 nuclei/4 animals, *t*-test: *p* < 0.0001; with inclusions: WT Syn foci volume 0.0976 ± 0.0047 μm^3^ N = 767 nuclei/4 animals, PLK2 KO Syn foci volume 0.1185 ± 0.0087 μm^3^ N = 482 nuclei/4 animals, *t*-test: *p* < 0.022). Scale bar 2 μm.

In order to study the potential direct effects of PLK2 genetic deletion on the immunohistochemical properties of Lewy pathology, we utilized the same PFF injection paradigm as in our *in vivo* imaging experiments (above) in a cohort of Syn-GFP/PLK2 WT, heterozygous KO and homozygous KO animals. Fixed cortical tissue from these animals was analyzed for Lewy body pathology, including phosphorylation, ubiquitination, and inclusion volume. To our surprise, unlike the decreased phosphorylated Syn-GFP levels we detected in presynaptic terminals (Fig. 2, *A* and *B*), PLK2 genetic deletion (either heterozygous or homozygous) showed no measurable effect on the level of Lewy pathology phosphorylation, ubiquitination, or on the volume of the somatic inclusions (Fig. 6*A*). Others have shown that PLK2 can regulate shuttling of alpha-synuclein between the nucleus and the cytoplasm (43). In addition, our previous work suggests that alpha-synuclein forms discrete, submicron-sized foci within cortical neuron nuclei that colocalize with established markers of DNA double-strand break (DSB) repair and that loss of alpha-synuclein from the nucleus in Lewy inclusion-bearing neurons may reduce this DSB repair function and contribute to cell death (44). For these reasons, we measured the levels of alpha-synuclein within nuclear foci in neurons, with and without Syn-GFP inclusions, in PLK2 WT and KO cortex. This analysis showed that the volume of alpha-synuclein foci, in both neurons with and without inclusions, was significantly larger in PLK2 KO animals compared with WT (Fig. 6*B*). This suggests that PLK2 kinase activity does modulate nuclear alpha-synuclein in a way that reduces the amount involved in DSB repair, that PLK2 KO can increase this amount, and that this increase may have beneficial effects in terms of promoting neuronal survival.

**Figure 7:**
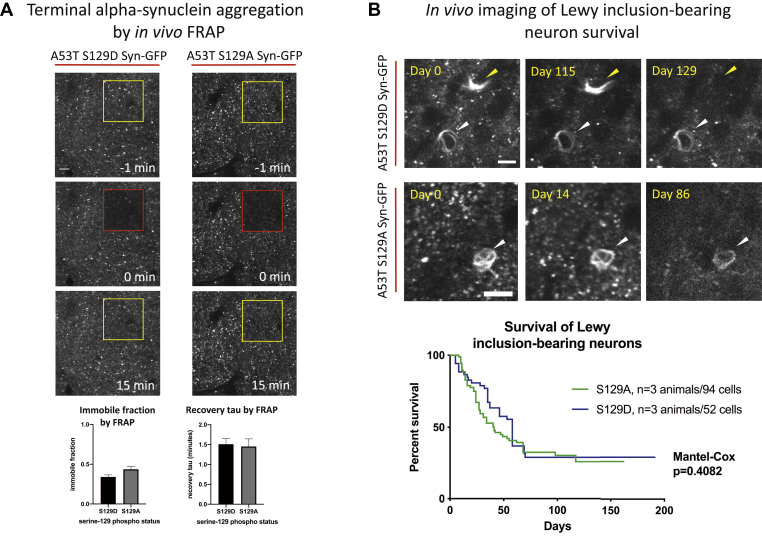
**Mutation of serine-129 to the phosphomimic aspartate, or unphosphorylatable alanine, does not alter *in vivo* measures of presynaptic alpha-synuclein aggregation or Lewy inclusion-bearing neuron survival.***A*, Top: *In vivo* multiphoton imaging from cortex in adult mice expressing A53T S129 (D or A) Syn-GFP after AAV intraventricular injection at postnatal day 0. Region of interest labeled with numerous Syn-GFP-positive presynaptic terminal puncta. Region of bleach for FRAP experiments shown within yellow square before, red square immediately after, and yellow square at indicated time point after the bleach pulse. Scale bar 10 μm. Bottom: The calculated immobile fraction of presynaptic terminal Syn-GFP at 15 min postbleach and recovery tau showed no significant differences between S129D and S129A expressing animals (immobile fraction: S129D = 0.340 ± 0.027, S129A = 0.436 ± 0.038, unpaired *t*-test, two-tailed *p* = 0.0530; recovery tau: S129D = 1.509 ± 0.141 min, S129A = 1.452 ± 0.191 min, unpaired *t*-test, two-tailed *p* = 0.8136). *B*, Top: Examples of A53T S129 (D or A) Syn-GFP Lewy inclusion-bearing neurons (*arrowheads*) followed *in vivo* in mouse cortex, one of which (*yellow arrowhead*) degenerates between 115 and 129 days. Scale bars 10 μm. Bottom: Group data shows no significant survival difference between S129D and S129A Syn-GFP inclusion-bearing neurons.

In order to study the direct role of alpha-synuclein phosphorylation at serine-129, independent of other changes that PLK2 KO can have due to potential non-kinase-related activity or kinase activity at other substrates, we generated mice that expressed Syn-GFP bearing the human disease-associated A53T mutation and mutation of serine-129 to either the phosphomimic aspartate (S129D) or the unphosphorylated residue alanine (S129A). These mutations would be expected to test whether phosphorylation at this site has any specific effects on presynaptic terminal or somatic Lewy body alpha-synuclein aggregation in our *in vivo* imaging paradigms. In order to obtain animals with sufficient expression of these Syn-GFP proteins in cortical neurons, we used an adeno-associated virus (capsid serotype 8, AAV8)-based strategy involving intraventricular injection of either A53T/S129D (or S129A) Syn-GFP AAV8 into mouse pups at postnatal day 0. As previously shown (45), this method produces robust and stable expression of proteins in cortical neurons and is easily amenable to our *in vivo* multiphoton imaging approaches. We first studied presynaptic terminal aggregation *in vivo* in these animals using our multiphoton FRAP approaches. Mutation of serine-129 to either S129D or A did not produce any detectible changes in the IF or recovery tau between the two groups (Fig. 7*A*), suggesting that mimicking the negative charge of phosphorylation at this residue in the S129D case does not alter presynaptic terminal Syn-GFP aggregation or binding to vesicle membranes *in vivo*. We next performed intracortical PFF injection into A53T/S129D (or S129A) Syn-GFP animals to induce Lewy pathology, followed by *in vivo* multiphoton imaging to measure the rate of cell death of Lewy inclusion-bearing neurons containing S129D (or S129A) Syn-GFP over the course of ∼160–190 days after inclusion formation. If serine-129 phosphorylation state affects neuronal survival of inclusion-bearing neurons, we should expect to measure differences in survival of neurons bearing these two kinds of (S129D or A) Syn-GFP inclusions. We did not detect any significant difference in the rate of cell death between these two groups of Lewy inclusion-bearing neurons (Fig. 7*B*). This suggests that serine-129 phosphorylation state does not directly influence cell death of Lewy inclusion-bearing neurons.

## Discussion

Our data show that endogenous mouse PLK2 constitutively phosphorylates transgenically expressed human alpha-synuclein at serine-129 in the presynaptic terminal in cortex, as previously reported for endogenous cortical mouse alpha-synuclein (32). In our experiments, acute pharmacologic inhibition with BI 2536 or PLK2 KO reduced levels of phosphorylated alpha-synuclein in the presynaptic compartment by ∼30–80%, depending on the condition and assay used. Although a substantial body of literature suggests that PLK2 is an important alpha-synuclein kinase (23, 26, 28, 30, 31, 32), potentially relevant in nonhuman primate (46) and human Lewy body disease tissue (26), genetic deletion of PLK2 in mice does not completely abolish endogenous mouse serine-129 phosphorylation (32). Our data are consistent with this previous report (32) and also suggest that other non-PLK2 kinases may be responsible for the remaining phosphorylated human alpha-synuclein levels that we measured in cortical terminals. Although PLK1 and PLK3 are potential candidates for mediating this remaining phosphorylation, previous work in PLK2 KO mice with BI 2536 treatment, or with PLK3 KO, did not produce further reductions in alpha-synuclein phosphorylation (32), strongly suggesting that another, non-PLK family of kinases, is important in phosphorylating terminal alpha-synuclein. As mentioned above, GRK and CK family members are potential candidates, and further work with pharmacologic inhibition and/or genetic deletion of GRK and CK family members will be important to test this directly.

In contrast to presynaptic terminal alpha-synuclein where PLK2 genetic deletion reduced phosphorylation, it had no effect on phosphorylated alpha-synuclein levels within Lewy inclusions. Although previous work suggested that PLK2 is important in phosphorylating aggregated alpha-synuclein *in vitro* and is specifically upregulated in human DLB brain (26, 28), our data suggest that its genetic deletion does not alter Lewy body phosphorylation in mammalian brain. It is likely that other, non-PLK2 kinases, either PLK1, PLK3, GRK, or CK family members are mediating Lewy body phosphorylation, and it will be interesting in future work to use pharmacologic and/or genetic strategies to test this.

Although PLK2 KO did not alter Lewy body phosphorylation, our *in vivo* multiphoton imaging paradigm detected a significant survival advantage of Lewy inclusion-bearing neurons on the PLK2 KO background, compared with PLK2 WT. This suggests that inhibition of PLK2 activity is neuroprotective, even if this may not be directly due to alterations in Lewy body phosphorylated alpha-synuclein levels. It is possible that reductions in phospho-synuclein levels in other non-Lewy body pools within the cell, such as nuclear or presynaptic terminal pools, are mediating this protective effect. In support of this possibility, we did detect a decrease in aggregation of presynaptic terminal alpha-synuclein as measured by *in vivo* multiphoton FRAP in the PLK2 KO animals. It has been suggested that specific presynaptic alpha-synuclein aggregate species drive neurodegeneration in disease (47, 48) and one mechanism by which PLK2 KO could have a protective effect is by lowering levels of these terminal aggregates. Alternatively, PLK2 has many non-synuclein targets and is important in spindle checkpoint signaling during the cell cycle in dividing cells (49) and in regulating the synaptic actin cytoskeleton in postmitotic neurons (50). To support the possibility that non-synuclein targets are important for mediating the neuroprotection in PLK2 KO animals, our experiments mutating serine-129, which showed no difference in survival between Lewy inclusion-bearing neurons containing S129D and A Syn-GFP, suggest that it is not alpha-synuclein phosphorylation that is causing the changes in neuronal survival we measure. Although there is evidence that mutation of serine-129 to aspartate, as we have done, (or glutamate) does not necessarily recapitulate all the effects of endogenous phosphorylation of the protein (51), we did not see an effect of the serine-129 to alanine mutation as well. The S129A alpha-synuclein form cannot be phosphorylated by PLK2, suggesting that the neuroprotective effect we measure with PLK2 KO is likely due to modulation of another, synuclein phosphorylation-independent pathway. Although the mechanism of the neuroprotective effect of PLK2 KO is yet to be determined, our work suggests that the development of PLK2-specific small-molecule inhibitors, several of which have already been produced (31), is a potentially promising strategy for slowing neurodegeneration in PD and DLB. This PLK2-specific strategy also has potentially distinct advantages related to toxicity. Currently available PLK inhibitors that have been used in humans all target PLK1, PLK2, and PLK3, and toxicity from PLK1 inhibition includes significant and dose-limiting myelosuppression (52). This makes developing PLK2-selective compounds important, since prolonged treatment times will likely be required when treating chronic neurodegenerative diseases, and therefore low-toxicity agents may be critical to provide large enough therapeutic windows and clinical benefit.

One potential mechanism by which PLK2 KO could be slowing the rate of cell death of Lewy inclusion-bearing neurons involves PLK2’s role in the nuclear-cytosolic transport of alpha-synuclein. PLK2 has been shown to promote shuttling of alpha-synuclein out of the nucleus and into the cytoplasm, potentially due to directly phosphorylating serine-129 and/or mediating phosphorylation of other targets (43). Our previous work suggests that alpha-synuclein has nuclear functions in the repair of DNA double-strand breaks (DSBs) (44). It is possible that loss of this function when cells form cytoplasmic Lewy inclusions, and nuclear alpha-synuclein levels are dramatically reduced (34), could contribute to programmed cell death. We detected an increase in the amount of nuclear alpha-synuclein present in DSB repair-associated foci in PLK2 KO cortical neurons compared with PLK WT, consistent with previous work outlining synuclein’s importance for nuclear-cytoplasmic shuttling (43). This increase in nuclear alpha-synuclein could ameliorate the defects in DSB repair present in Lewy inclusion-bearing neurons (44) and promote their survival. Our data is also consistent with another report demonstrating that increasing nuclear alpha-synuclein levels can be neuroprotective (53).

In summary, our data suggest that PLK2 is not a Lewy body kinase, but that it does contribute to the phosphorylation of alpha-synuclein within the presynaptic nerve terminal. Although preventing or mimicking serine-129 phosphorylation does not alter the rate of cell death of Lewy inclusion-bearing neurons *in vivo*, PLK2 genetic deletion does provide relative protection from cell death of these vulnerable neurons, and selective pharmacologic inhibition of PLK2 may represent a promising strategy for modifying disease progression in PD, DLB, and related disorders.

## Experimental Procedures

### Animals

Animals were housed by OHSU’s Department of Comparative Medicine in a light–dark cycle, temperature and humidity-controlled vivarium, and maintained under *ad libitum* food and water diet. All experiments were approved by the OHSU IACUC. All experiments were performed in accordance with the relevant guidelines and regulations, and every effort was made to minimize the number of animals used and their distress. Syn-GFP animals have been previously described (PDNG78 (33), and PLK2 KO animals were obtained from The Jackson Laboratory (stock # 017001). Both male and female mice were used, but no significant differences were detected based on sex, so both groups were combined in the analyses.

#### Intraventricular AAV8 viral injections

We created viral constructs of A53T Syn-GFP containing point mutations in alpha-synuclein at serine-129 causing either an alanine (TCT > GCT) or aspartic acid (TCT > GAT) amino acid change (constructs gift of Pamela McLean). These constructs were cloned into the self-complementary AAV8 viral vector pTRS-KS/CBh-GFP (from the National Gene Vector Biorepository, Indianapolis, IN), under transcriptional control of the CBh (Chicken Beta Actin Short) promoter, using the restriction enzyme sites AgeI and ApaI. The 30 bp linker sequence between alpha-Syn & enhanced GFP is GGTACCGCGGGCCCGGGATCCATCGCCACC. The final scAAV8-CBh-A53T-S129A-Syn-GFP and scAAV8-CBh-A53T-S129D-Syn-GFP viruses were made by the Gene Transfer Vector Core at Massachusetts Eye and Ear Infirmary, Boston, MA. Intraventricular injection into P0 neonates was done following the free-hand protocol (45). We injected 2 μl of undiluted virus into each lateral ventricle at titers ∼1E12 genomic copies per milliliter. The only deviation from this published protocol is that we did not dilute virus in trypan blue.

### Western blotting

Brains from male and female mice 3–4 months of age were dissected immediately postmortem, and olfactory bulbs and cerebellum were removed. Remaining brain tissue was prepared for western blot analysis *via* use of Syn-PER Synaptic Protein Extraction Reagent (Thermo Fisher) using the manufacturer’s protocol, resulting in both a synaptosomal and a cytosolic fraction. Samples were run on a 4–12% Tris-glycine gels (Invitrogen) for 2 h and 45 min at 80 V. Gels were transferred onto Immobilon-FL membranes (0.45 μm pore; Millipore) at 25 V for 18 h at 4 °C. Membranes were blocked with Odyssey blocking buffer (Li-COR Biosciences) for 1 h at room temperature. Membranes were incubated overnight with primary antibodies (alpha-synuclein: 1:1000, Syn-1, BD Biosciences; serine-129 phospho-alpha-synuclein: 1:1000, EP1536Y, Abcam; GAPDH: GAPDH, 1:10,000, Millipore). A near infrared fluorescent-labeled secondary antibodies (1:5000; IR800 & IR700; LI-COR Biosciences) were used, and quantification was done with an Odyssey CLx infrared imaging system (LI-COR Biosciences) and ImageJ/Fiji (NIH).

### Lewy pathology formation

Two-to three-month-old mice were injected with mouse WT sequence PFFs. Injections were done according to our published protocols for PFF generation and preparation (Luk *et al*., 2012b) and cortical injections (34). Briefly, 2.5 μl (2 mg/ml) of freshly sonicated PFFs was injected into right hemisphere primary sensory-motor cortex in isoflurane (1% to 2%)-anesthetized animals.

### Immunohistochemistry

Whole brains were dissected from male and female mice, and the cerebellum and olfactory bulbs were removed. Brains were divided into two along the midsagittal plane. Both hemispheres were placed in plastic scintillation vials with 4% paraformaldehyde and fixed for 1 h, 150 W, at 30 °C using a BioWave (Pelco) microwave fixation system. Hemispheres were postfixed in 4% paraformaldehyde overnight at 4 °C and then stored in PBS containing sodium azide (0.05%). Fixed hemispheres were mounted on a Leica VT1000 S Vibratome and sagittally sectioned into 50 μm slices. Slices were stored in PBS containing sodium azide at 4 °C. Then 50 μm fixed brain slices were washed three times in PBS and incubated in blocking buffer (0.1% Triton-X, 10% goat serum) for 2 h while shaking in the dark. Primary antibodies (alpha-synuclein: 1:500, Syn-1, BD Biosciences; serine-129 phospho-alpha-synuclein: 1:667, 81A, Biolegend; ubiquitin:1:200, Z0458, Dako) were incubated with shaking in the dark overnight at 4 °C. Tissue was washed in PBS five times for 30 min each. Secondary antibodies goat anti-mouse or goat anti-rabbit Alexa Fluor 647 (Invitrogen) were diluted to 1:2000 and subjected to incubated shaking in the dark overnight at 4 °C. Tissue was washed in PBS five times for 30 min each at room temperature. Brain slices were mounted on slides in CFM-2 (Ted Pella), sealed with CoverGrip (Biotium), and allowed to dry overnight in the dark. For confocal imaging, images were acquired on a Zeiss Elyra PS.1 confocal microscope with a Plan-Apochromat 63x/1.40 oil objective. Laser powers of 1–5% were used to acquire z-stacks through 2–4 regions of cortex per tissue section. Imaris (Bitplane, Oxford Instruments) or ImageJ/Fiji (NIH; (54); imaging software was used to analyze the IHC data.

### *In vivo* multiphoton imaging

Cranial window surgery, imaging, and analysis were done using the same protocol as we have previously published (34, 36) in isoflurane-anesthetized animals at 3–5 months of age (2 months after PFF injection, in animals that received this treatment). We used a Zeiss LSM 7MP multiphoton microscope outfitted with dual-channel BiG (binary GaAsP) detectors and a Coherent Technologies Chameleon titanium-sapphire femtosecond pulsed laser source (tuned to 860 nm for imaging Syn-GFP). Zeiss Zen 2011 image acquisition software was used. Images were analyzed with ImageJ/Fiji NIH (54) to obtain mean fluorescence values in relevant ROIs over time. FRAP data were analyzed in Prism 6 (GraphPad) to obtain single exponential fits to the recovery time course and the immobile and mobile fractions.

### Behavioral analysis

Behavioral analysis was performed on 4–5 month-old animals (N= WT/PLK2 WT 11, WT/PLK2 Het 7, Syn-GFP/PLK2 WT 15, Syn-GFP/PLK2 Het 6, Syn-GFP/PLK2 KO 11 animals). Open field, novel object, rotarod, Morris water maze, contextual and cued fear conditioning behavior measures were performed as previously published (55).

## Statistics

All data are reported as the mean ± SEM unless otherwise noted. Numbers and statistical tests used are reported in each relevant section.

## Data availability

All data are presented in the article and the supplementary information.

## Conflict of interest

The authors declare that they have no conflicts of interest with the contents of this article.
