# Supplementary material for: Genetic deletion of Polo-like kinase 2 reduces alpha-synuclein serine-129 phosphorylation in presynaptic terminals but not Lewy bodies
Source: J Biol Chem. 2021 Jan 9;296:100273. doi: 10.1016/j.jbc.2021.100273 (PMC7948797; doi:10.1016/j.jbc.2021.100273)

Supplementary information

Supplementary Figure 1. Polo-like kinase 2 genetic deletion does not alter many behavioral measures. (**A**) Open field measure of anxiety (% time spent in the center of the field) are not significantly different between WT and Syn-GFP animals on a PLK2 WT (+/+), Het. (+/-), or KO (-/-) background (repeated measures ANOVA: Syn-GFP p=0.667, PLK2 p=0.843). (**B**) Novel object exploration measure (% time exploring) showed significant differences between novel and familiar object, but not between WT and Syn-GFP or different (WT, Het., KO) PLK2 background groups (two-way ANOVA: object p=<0.0001, group p=0.999; post-hoc Sidak's tests: WT p=0.0007, PLK2 Het./WT p=0.0007, WT/Syn-GFP p=0.0001, PLK2 Het./Syn-GFP p=0.9836, PLK2 KO/Syn-GFP p=0.0294). (**C**) Rotarod measure (latency to fall) showed no significant differences between WT and Syn-GFP animals on the different PLK2 backgrounds (repeated measures ANOVA: Syn-GFP p=0.351, PLK2 p=0.399). (**D**) Morris Water Maze test of spatial platform memory at 24hr measure (% time in quadrants) showed no significant differences between WT and Syn-GFP animals on PLK2 WT and KO backgrounds, although animals on the PLK2 Het. background showed reduced spatial memory (two-way ANOVA: ** p<0.01, **** p<0.0001). (**E**) Fear conditioning with spatial contextual cues measure (% time freezing) showed no significant differences between WT and Syn-GFP animals but did show a significant effect of PLK2 genotype (repeated measures ANOVA: Syn-GFP p=0.682, PLK2 p=0.033; post-hoc Sidak's tests: PLK2 KO vs. Het. p=0.035). (**F**) Fear conditioning with tone cues measure (% time freezing) showed no significant differences between WT and Syn-GFP animals on the different PLK2 backgrounds (repeated measures ANOVA: Syn-GFP p=0.318, PLK2 p=0.455).

Supplementary Figure 2. *In vivo* formation of Lewy inclusion and measures of inclusion aggregation. (**A**) Top: *In vivo* multiphoton imaging from cortex in a Syn-GFP (PLK2 WT) mouse after PFF injection showed growth of individual somatic Lewy inclusion over the course of 15 days. Image inverted so that high Syn-GFP signal shown as black in the interest of clarity. Scale bar 10 μm. Middle & Bottom: Same inclusion shown at a lower Z-planes, each 2 μm apart in the Z-axis. (**B**) Top: *In vivo* multiphoton image of Syn-GFP mouse cortex with a somatic inclusion studied by FRAP in order to measure aggregation. Region undergoing FRAP

(rectangle) shown before (yellow), immediately after (red) and for 5 minutes after bleach pulse (yellow). Bottom: Group data showed no difference in somatic Lewy inclusion aggregation state as measured *in vivo* by FRAP immobile fraction at 5 minutes in PLK2 WT and KO animals (WT=1.00±0.03, KO=0.99±0.01, unpaired t-test, two-tailed p=0.7617).

Supplementary Figure 3. Frequency of *in vivo* multiphoton imaging in Syn-GFP mice does not alter rate of Lewy inclusion-bearing neuron cell death. Group data showing that Syn-GFP inclusion-bearing neurons died at similar rates regardless of whether they were imaged infrequently (3 times) or frequently (17 times) over a ~160 day period, suggesting that cell death is not due to phototoxicity from chronic *in vivo* imaging.

Supplementary Figure 1

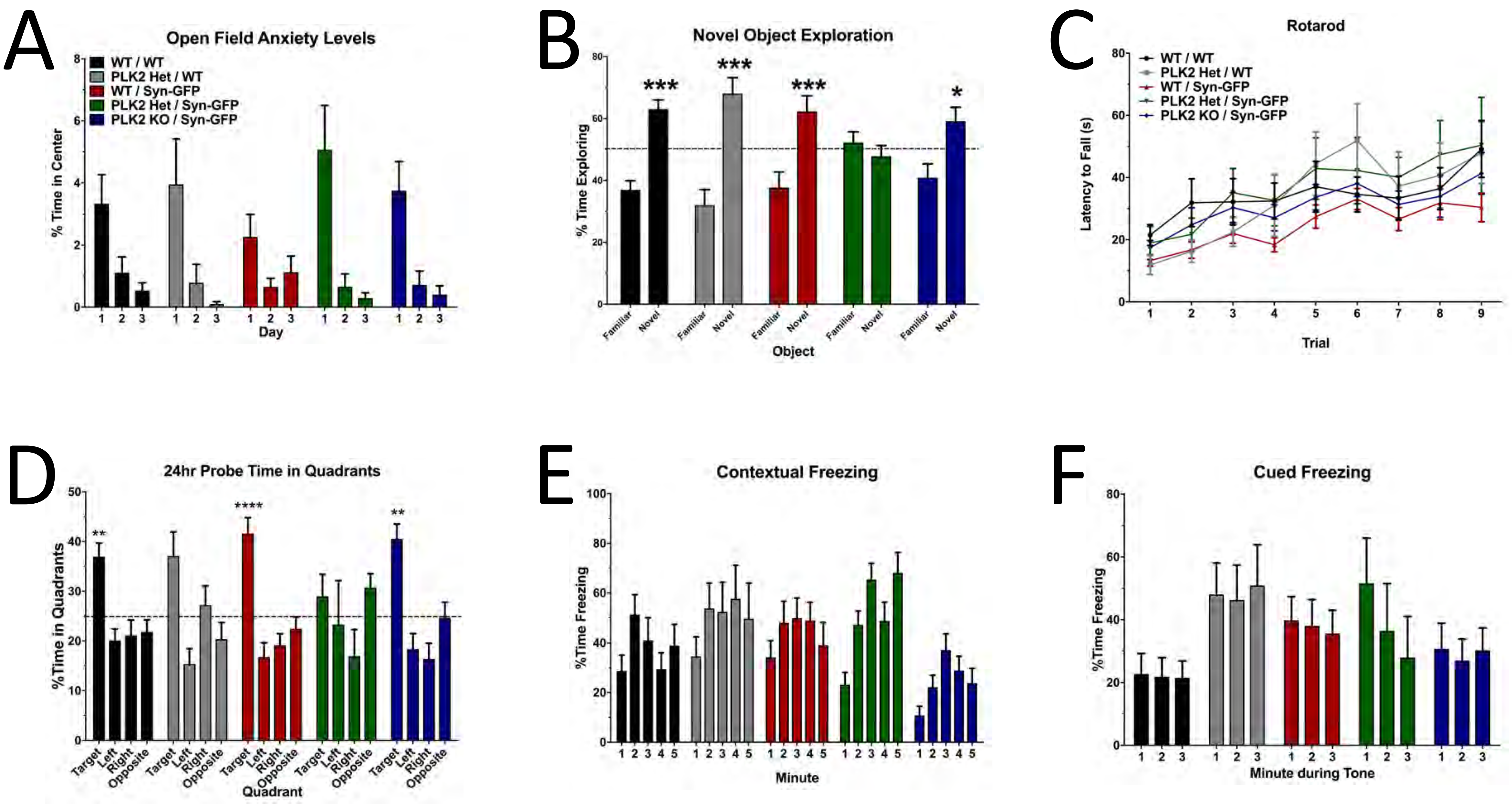

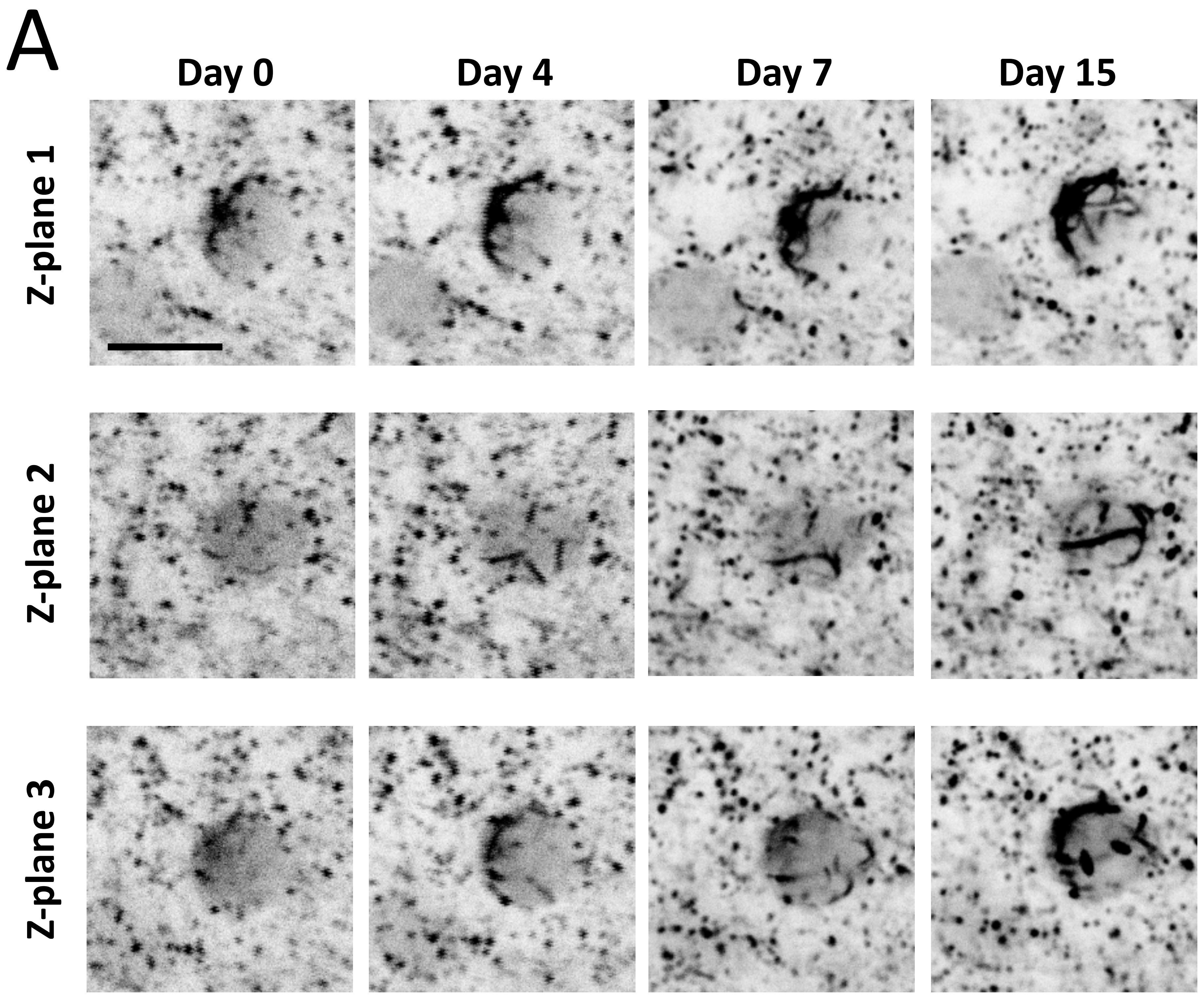
A
Day 0
Day 4
Day 7
Day 15
Z-plane 1
Z-plane 2
Z-plane 3

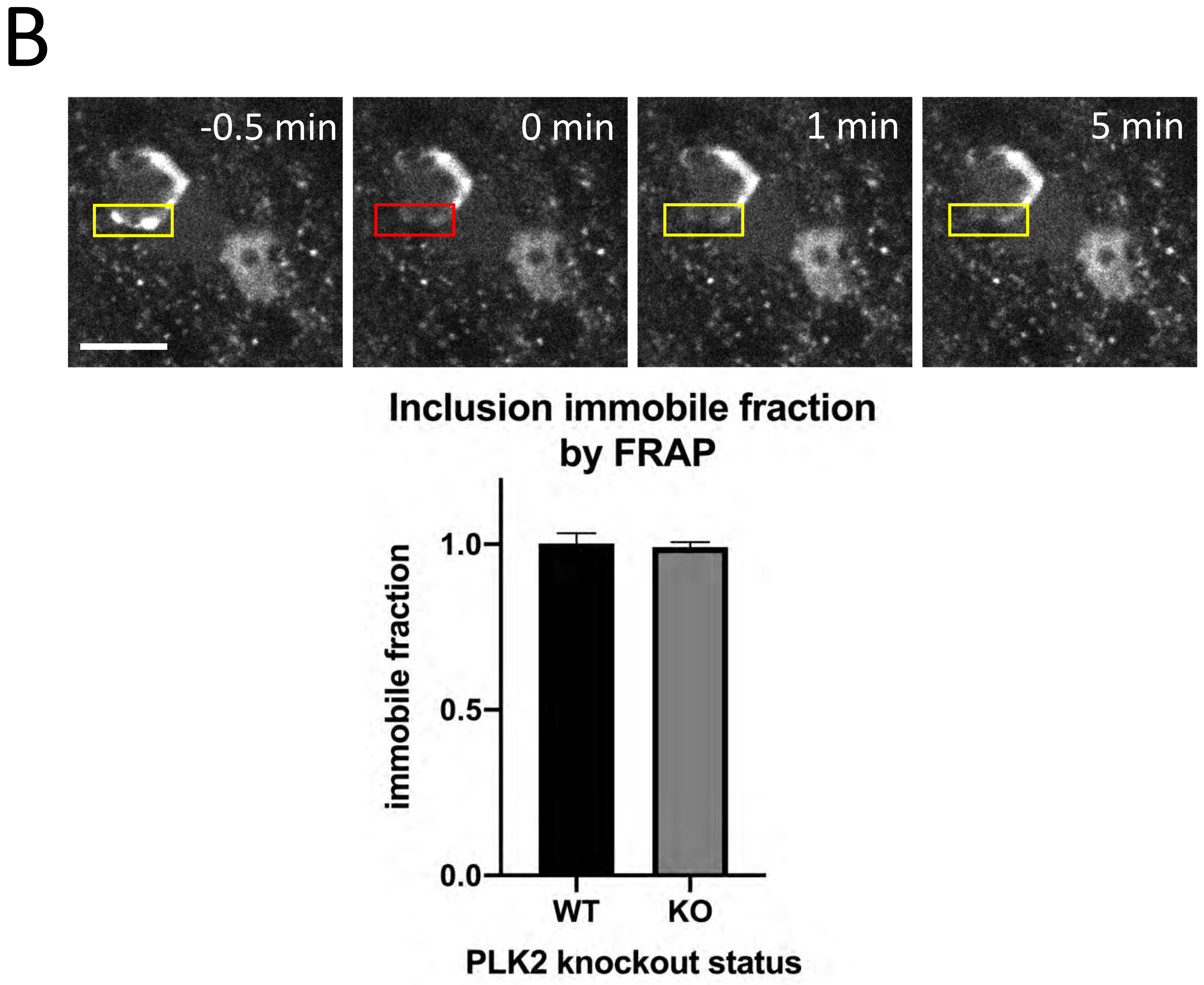
B
-0.5 min
0 min
1 min
5 min
Inclusion immobile fraction by FRAP
immobile fraction
1.0
0.5
0.0
WT
KO
PLK2 knockout status

Supplementary Figure 3

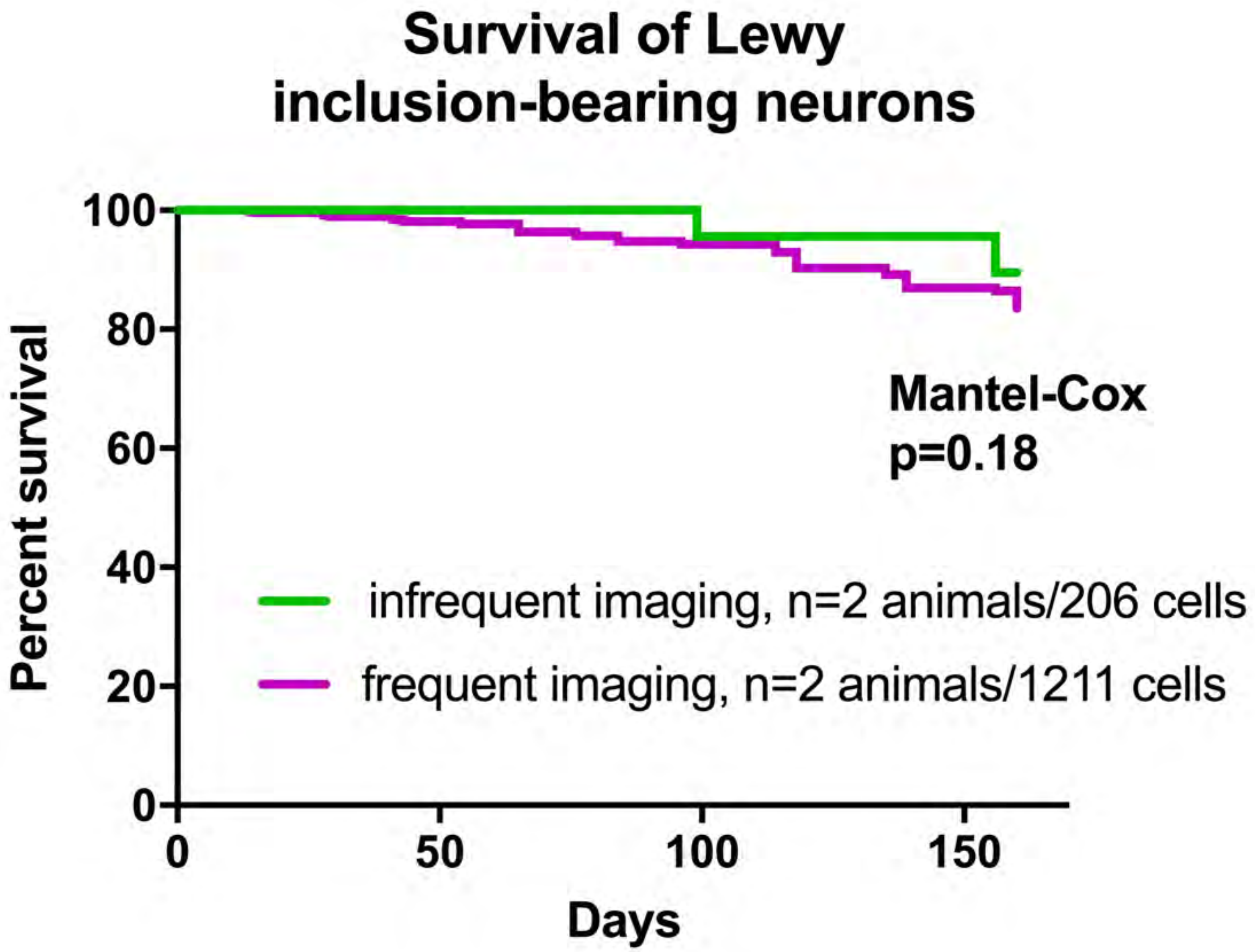

Supplement: Figures S1–S3 [file mmc1.pdf]
